# Is the Division of Diseases into General and Local Consistent with Truth?

**Published:** 1813-09

**Authors:** 


### *Is the Division of Diseases into general and local consistent with Truth?*

*To the Editors of the Medical and Physical Journal.*

GENTLEMEN,

UPON reading Dr. Clutterbuck's treatise on fever, published about six years ago, some remarks occurred to me, which I noted at the time, and now submit to you for insertion in the Medical Journal. As I am actuated by no other motive than that of professional improvement, I trust you will not disregard my opinions because they appear unsupported by a name, and though differing from those of Dr. Clutterbuck, contain nothing derogatory to his character as an eminent physician, and learned teacher.

In his able work on fever, Dr. Clutterbuck denies the existence of any general or universal disease, and supports this opinion with a variety of facts and ingenious reasoning. He states, "that, strictly speaking, all diseases are in their origin local, or affections of some particular parts or organs, and never of the entire system." Again, "a disease can only, in strictness, be termed general or universal, when it affects every part of the system at once. But there are evidently none such. The whole system may, indeed, be weakened, and all its actions be consequently diminished, as by loss of blood; but such a state, if it affect all parts equally, is not a disease, though it perhaps strongly predisposes to it. Something more is wanted to constitute morbid action. Under such a state of general weakness, the functions may continue to be carried on, though less vigorously than before, and until one or more of these become deranged or interrupted, or until some uneasy sensation is induced, disease can hardly be said to exist."

The learned author then proceeds to examine the Phlegmasiæ, the Hæmorrhagiæ, and the Profluvia, orders of the first class Pyrexiæ of Cullen; which he determines to be essentially local affections; leaving the remaining orders of that class, the Febres and Exanthemata, for future consideration. With equal dispatch and facility, he disposes of the three other classes.

Of asthenic diseases, we are informed "that debility, though it may give a predisposition to disease, is of itself rarely, if ever, either the proximate or the occasional cause." "It is in general an effect only, and indicates nothing certain with regard to the cure." The practice of giving tonic and stimulant remedies, and administering rich diet and nutritious food in cases of general weakness, is ridiculed and pronounced to be mischievous; because "by increasing the topical affection, they often tend to depress the energy of the system still further, instead of rousing it." As to the opposite state of the system, the inflammatory or sthenic diathesis, with excess of action, the author doubts its existence altogether.

In support of this opinion, it is alleged, "that an increase or diminution of action in one part generally induces a contrary mode of acting in others; and lastly, that the causes exciting disease, all of them, produce peculiar or specific effects; it might naturally be expected that diseases would always be partial or local at their commencement." The Phlegmasiæ, the Hæmorrhagiæ, and the Profluvia, of Cullen's first class Pyrexia, are essentially local, as they are often present without any general disorder of the system; and when a febrile state of the system (which is sometimes the case) does precede these local affections several days, they are to be considered either as complications of proper fever with topical inflammation, or of a conversion of the former into the latter. In this way the author briefly disposes of every disease. I will now attempt to inquire how far his opinion is correct.

The first point to be determined is, what may be regarded as a general disease and what local. The author denies a disease is general, unless every particular function and organ of the body be deranged, and undergoing morbid action; and this immediately after the exciting cause or power is applied. He has here taken a strong position, and if we admit this as the basis of his reasoning, any attempt to dislodge him must be fruitless; for, though we may conceive that every part of the body is undergoing morbid action, it is is very difficult to prove it; and dissection after death not unfrequently brings diseased parts to view, which had given no previous symptom of having been the subject of disease. The author himself has informed us, that "a disease may consist merely in morbid action, occasioning derangement of functions, without any perceptible alteration of the structure or organization of the part affected; and in such cases no insight into the seat or nature of the disease can be derived from dissection after death."

May we not consider a disease to be general, when from the very commencement a patient complains of universal indisposition, without being able to refer to any particular part or organ as the seat of the disorder? In this state we find the patient affected with general lassitude, cold shivering, headach, or sense of heaviness, soreness over the body, depression of mind, anorexia, and sometimes nausea; the eyes have lost their lustre, the whole countenance appears shrunk and void of animation; in a few hours, or sometimes longer, the heart pulsates quicker, and with greater or less force; the heat of the body is increased, and sometimes perspiration breaks out. At this early period, it is impossible for the most acute and experienced practitioner to pronounce what the future disease is to be; if he sees his patient within six, twelve, or even eighteen hours from the first moment of his feeling indisposed, the practitioner often shall not be able to say whether the complaint will be simply fever, inflammatory sore-throat, peripneumony, measles, acute rheumatism, or in fact any disease accompanied by pyrexia and general indisposition. Are we to regard this as a fair case of disease? If we are, I ask where is the locality of the affection? If the advocates for all diseases being local, deny that it is disease; they may call it predisposition, but where is the distinction, for many cases of acknowledged disease continue and go through their course with no other symptoms? It is not unusual to meet with a complaint, which, from its extreme short duration, has been called Ephemera, and Diaria. It attacks the patient suddenly without any apparent cause. The symptoms are pain in the head, prostration of strength, and chilliness; in the course of the day, the pulse becomes very frequent, respiration quick and short; the surface of the body red, and the face is sometimes slightly swelled; and in the course of the same day, this complaint terminates with sweating; and I have the authority of Sauvages and Lommius for asserting that it is not protracted beyond two or three days at most. Surely this will not be called only predisposition; and if it is to be classed under the the great head local disease, it would be difficult to point out the organ primarily affected.

I think I have now pretty clearly made out that a disease may be general, though we have no direct evidence of each particular organ being deranged; and if, from this enumeration of symptoms, it appears to be so, by investigating as much as we are able the phenomena of febrile disease, its mode of acting on the body, and the means by which it is removed, perhaps we shall be still more confirmed in that opinion. In doing this, however, I shall confine myself, for the sake of brevity, to the consideration of one or two of the febrile diseases; for the same reasoning that will apply to one will be applicable to another. And here it may be proper to observe, that though the author professed in the beginning of his work to demonstrate in a future part of it that the Febres and Exanthemata of Cullen's first class Pyrexiæ, were decidedly local diseases, he has entirely omitted to prove his assertion, resting satisfied with his intention. Perhaps it might be easier to prove that idiopathic fever is a general disease; but I will take Peripneumony as an instance of what I consider a general disease. It begins with the usual symptoms of fever, and the pyrexia is often present several hours before pain is felt in the chest, or cough is excited, the pulse being frequent and strong; cough, dyspnœa, and pains in the side and thorax, shortly succeed; the expectoration is often streaked with blood; but sometimes, particularly in the beginning, the cough is dry. These are the leading symptoms, varying, of course, in different constitutions, habits of life, age, and season.

The causes of this complaint are chiefly the vicissitudes of heat and cold experienced in the winter or spring seasons. But millions of people at these seasons of the year are daily exposed to every cause of peripneumony without being subject to the complaint. Now I contend that, was it a local affection, were the lungs independent of the system, performing their functions unaided and uninfluenced by any other organs, they would, when exposed to the exciting cause, at all times be liable to the disease in question; but they are not. They are immediately connected with the whole system by nerves, blood-vessels, and absorbents; and before they can be affected as they are in peripneumony, the whole system must be subjected to some change or action, which, if not morbid, is so nearly allied to it, that I cannot distinguish between the two states. Who are principally the subjects of acute peripneumony? Not the feeble; not those of a spare habit and lax fibre: it is the robust, the strong, the free-livers, men from twenty-five or thirty, to forty years of age.

Whenever

Whenever the quantity of blood is increased by excess in eating or drinking, by indolence and inactivity, and a general fulness of the vascular system is present; or where its velocity is much accelerated by great exertion in running, or labor; in such a state, particularly where there is a disposition to plethora, and which, in spite of Dr. Clutterbuck's objections, I consider as a sthenic or inflammatory diathesis; in such a state, I say, the causes of peripneumony, viz. the vicissitudes of heat and cold being applied, the symptoms may appear pretty much in the order before stated. The whole system is undergoing morbid action, and the lungs are merely affected as forming part of that system; and I can assign no reason why the disease, under similar circumstances, might not have been acute rheumatism, as well as peripneumony; the two affections are frequently blended together. A strong proof of the system, in either case, being generally affected, is, that the pain frequently moves to different parts; nor does it entirely cease till the general affection of the system is removed. This brings us to the cure; which is so well known to depend entirely upon remedies acting on the whole system, that it scarcely merits notice in this place. Large and repeated general bleeding, and purgatives, not administered so much with the view of merely evacuating the intestines, as to lower the inflammatory state of the system, to effect which every means within the power of medicine is indicated; and when this is happily accomplished, the effect ceases; the patient has nothing to contend with but debility, and perhaps in some cases cough may remain a little longer. If blisters are used, they are merely considered as adjuncts, and as such unquestionably are serviceable. The cough also must be attended to.

In a general disease, requiring general treatment, there may be parts of the system more affected than some other parts, and which, independent of the remedies given to act on the system generally, demand some particular applications, for the more speedy relief of the patients. But a general disease can only be cured by remedies which act universally on the system.

If the preceding observations are correct, I think it may fairly be laid down as a general proposition, that where local affections, or organs undergoing morbid action, are preceded by universal indisposition, the disease may in all cases be regarded as a general disease, to be cured only by general treatment.

To enumerate more instances of general disease, would occupy too much space in your Journal: I shall now, therefore, merely attempt to show what is meant by a local disease; disease; and offer some remarks upon the importance of discriminating between the two affections. The first characteristic difference between local and general disease, is, that the latter is always preceded by predisposition, the former never; without predisposition there can be no general disease. Thus we occasionally meet with instances where the small-pox cannot be communicated, and it is very properly stated that in such cases there is no predisposition; again, the cow-pox will in general be found to destroy this predisposition. A local disease may be produced by wounds, acrid substances occasioning erosion of parts, in short, whatever produces solution of continuity. This is the simplest species, and belongs to the province of the surgeon. When, however, organs highly sensible become affected, and inflammation is produced, the symptoms are often violent and dangerous; and perhaps it was from considering some cases of this kind which induced Dr. Clutterbuck to regard every disease as local. In gastritis or inflammation of the stomach, a patient immediately preceding the attack is in perfect health; he accidentally or purposely swallows some highly stimulating matter, as corrosive sublimate, pins, ground glass, ardent spirit, &c. &c. and inflammation of the organ speedily ensues; and from the high sensibility of the part, the system becomes generally affected; but this is evidently secondarily. In enteritis, or inflammation of the intestines, the same; and so it is with every acutely sensible organ where matter sufficiently offending is applied. In organs less sensible, or where the exciting cause is less potent, the morbid action is slow, and for a long time confined to the part first affected; thus we often meet with diseases of some of the glands which have existed for months without producing any constitutional disease whatever.

Perhaps a great source of error in the consideration of local and general disease, is, that the same organ is often diseased and its functions deranged very similarly in both cases; this is particularly observable in the stomach, an organ extremely subject to disturbance in its functions. In almost every general disease it is affected from the very beginning, or at least its interrupted action is one of the leading symptoms. Again, it is very frequently primarily affected, and subjected to chronic complaints, which, had the cause applied been sufficiently strong, would have been acute.

To discriminate then between local and general disease is essential to the practitioner. It gives confidence to his practice, and enables him more correctly to form his judgment, and estimate the probable result of the case. When a general ral disease is very speedily accompanied by urgent local affections, usually before we see our patient, it is surely of some consequence to be aware that notwithstanding the violence of the local symptoms, and the distress experienced from the suffering of parts, it is the general state of the system to which we are to direct our chief attention, and apply our remedies. Again we are called in when the whole constitution is suffering from the disease of some organ; and unless from our acuteness of perception, and the previous history of the attack, we discover the real nature of the complaint, we act completely in the dark; and consequently prescribe with the feebleness of timidity, or the temerity of ignorance.

I therefore regard the division of diseases into general and local, as being highly momentous and essentially useful, whilst it strictly accords with nature. What can the advocate of the opposite side of the question gain by his discovery? Though he does consider every disease in its origin to be local, when he comes to treat diseases which are now regarded as general from their commencement, he must prescribe as if treating a general disease; and he loses a very powerful assistant to his judgment, and I fear will be much more liable to error and misconception.

Having now very hastily and imperfectly stated what occurred to me upon the subject immediately after perusing Dr. Clutterbuck's work on fever, I cannot conclude without noticing two arguments upon which he chiefly depends in support of his hypothesis. Though he regards the phlegmasiæ as local diseases, because they are often present without any general disorder of the system, he is fully aware that they are often preceded by febrile action and general indisposition; such cases he contends are cases of proper fever, complicated with topical inflammation; but proper fever, he also contends, is topical inflammation of the brain; therefore, peripneumony must be inflammation of the brain, combined with topical affection of the lungs. So also, cynanche tonsillaris, measles, &c. being preceded by fever, must be regarded as inflammation of the brain, with topical affection of the throat, &c. Thus the very explanation of his doctrine confutes itself. The only other opinion which I shall trouble you with commenting upon, is, in my apprehension, a very mischievous one, and contrary to truth. The author, in his zeal for establishing the locality of disease, will not admit any general asthenic state of the system; or, if it is present, he contends that it is a consequence of some topical complaint, or diseased state of some particular organ, and by giving what are termed strengthening remedies, we increase increase the complaint by exciting greater action in the morbid part. Unquestionably in some instances this is true; but surely the author must have seen numerous cases of illness where the whole constitution appears to be sinking under debility; where there is extreme languor, feebleness, want of vigor, and general depression; where, in short, the universal frame seems on the verge of dissolution; and where, except general pains in the extremities, back, loins, &c. indeed over all the frame, (which is frequently the case in extreme debility,) no local affection whatever is either complained of or discovered. And how is this state removed, but by the very means which the learned doctor so decidedly condemns? by stimulant and tonic remedies, nutritious diet, pure air, and by every method which affords to the system a grateful and pleasant stimulus.

Having thus freely offered my sentiments upon this interesting subject of inquiry, I hope it will be considered that I have intended nothing personal in my remarks. It is the doctrine merely which I wished to discuss, and I am truly happy in being able to offer a public tribute of my respect for the worthy author, with some of whose opinions I have had occasion to differ; whatever may be our sentiments of his peculiar theory, the work in which it is developed contains much valuable information and ingenious disquisition; and no man capable of understanding it, can rise from its perusal without being benefited by his labor.

